# Reck enables cerebrovascular development by promoting canonical Wnt signaling

**DOI:** 10.1242/dev.136507

**Published:** 2016-03-15

**Authors:** Florian Ulrich, Jorge Carretero-Ortega, Javier Menéndez, Carlos Narvaez, Belinda Sun, Eva Lancaster, Valerie Pershad, Sean Trzaska, Evelyn Véliz, Makoto Kamei, Andrew Prendergast, Kameha R. Kidd, Kenna M. Shaw, Daniel A. Castranova, Van N. Pham, Brigid D. Lo, Benjamin L. Martin, David W. Raible, Brant M. Weinstein, Jesús Torres-Vázquez

There were errors published in Development **143**, 147-159.

In the Funding section, grant [RSG-14045-01-DDC] should have been attributed to the American Cancer Society and not to the American Chemical Society. Tables S1 and S2 were missing from the supplementary data. These errors have been corrected online.

We apologise to the authors and readers for these mistakes.

